# Identification of Optogenetically Activated Striatal Medium Spiny Neurons by *Npas4* Expression

**DOI:** 10.1371/journal.pone.0052783

**Published:** 2012-12-26

**Authors:** Asim K. Bepari, Hiromi Sano, Nobuaki Tamamaki, Atsushi Nambu, Kenji F. Tanaka, Hirohide Takebayashi

**Affiliations:** 1 Department of Morphological Neural Science, Graduate School of Medical Sciences, Kumamoto University, Kumamoto, Japan; 2 Division of Neurobiology and Anatomy, Graduate School of Medical and Dental Sciences, Niigata University, Niigata, Japan; 3 Division of System Neurophysiology, National Institute for Physiological Sciences, Okazaki, Japan; 4 Department of Physiological Sciences, Graduate University for Advanced Studies (Sokendai), Okazaki, Japan; 5 Department of Neuropsychiatry, Keio University School of Medicine, Tokyo, Japan; 6 PRESTO, Japan Science and Technology Agency (JST), Saitama, Japan; INSERM/CNRS, France

## Abstract

Optogenetics is a powerful neuromodulatory tool with many unique advantages to explore functions of neuronal circuits in physiology and diseases. Yet, interpretation of cellular and behavioral responses following in vivo optogenetic manipulation of brain activities in experimental animals often necessitates identification of photoactivated neurons with high spatial resolution. Although tracing expression of immediate early genes (IEGs) provides a convenient approach, neuronal activation is not always followed by specific induction of widely used neuronal activity markers like *c-fos*, *Egr1* and *Arc*. In this study we performed unilateral optogenetic stimulation of the striatum in freely moving transgenic mice that expressed a channelrhodopsin-2 (ChR2) variant ChR2(C128S) in striatal medium spiny neurons (MSNs). We found that in vivo blue light stimulation significantly altered electrophysiological activity of striatal neurons and animal behaviors. To identify photoactivated neurons we then analyzed IEG expression patterns using in situ hybridization. Upon light illumination an induction of *c-fos* was not apparent whereas another neuronal IEG *Npas4* was robustly induced in MSNs ipsilaterally. Our results demonstrate that tracing *Npas4* mRNA expression following in vivo optogenetic modulation can be an effective tool for reliable and sensitive identification of activated MSNs in the mouse striatum.

## Introduction

Optogenetic technology is rapidly fostering the study of brain functions through delineation of complex neuronal networks [1]. Incorporation of microbial light-activated regulators of transmembrane conductance in experimental animals allows manipulation of electrical activity of the target tissue with the millisecond temporal precision even in freely moving animals. When functional light-activated channel proteins are expressed in neurons both gain-of-function and loss-of-function studies are possible to modulate animal behaviors and to infer causal relationship between the illuminated neurons and the resultant cellular or behavioral changes in physiological or pathophysiological conditions. For instance, Brown et al. (2010) found that direct optical activation of dopamine (DA) neurons of the mouse ventral tegmental area (VTA) was sufficient to drive redistribution of AMPA receptors [2]. Using an optogenetic approach Deisseroth and colleagues (2009) demonstrated that selective high-frequency stimulation of afferent axons projecting to the subthalamic nucleus robustly ameliorated the disease symptoms in a rodent model of Parkinson's disease (PD) [3].

One of the big issues during in vivo optogenetic manipulation is that the illuminated cells are inevitably heterogeneous in terms of intensity of the incident light [1] and it is difficult to identify photoactivated cells precisely. This limitation calls for additional measures for sensitive detection of photoactivated cells with high spatial resolution. One convenient approach is the visualization of activated neurons by tracing induction of immediate early genes (IEGs) such as *c-fos*, *Egr1* and *Arc* [4]–[7]. Nevertheless, previous studies indicated that *c-fos*, the most widely used IEG, is not a universal marker for neuronal activation and IEGs may be differentially induced depending on the neuronal population and/or the stimulus. For instance, Isogai et al. (2011) found that *Egr1*, but not *c-fos*, was induced robustly in the mouse vomeronasal organ following sensory stimulation [8]. Though several types of drugs of abuse can induce c-fos in the striatum [9], [10], an atypical antipsychotic drug clozapine was found to induce *Egr1* but not *c-fos* mRNAs in the rat striatum [11]. Using in vivo light stimulation followed by in situ hybridization of activity markers here we show that the neuronal IEG *Npas4* can identify photoactivation of striatal medium spiny neurons (MSNs) more reliably compared to other commonly used IEGs like *c-fos*, *Egr1* and *Arc*.

## Results

### In vivo optical stimulation of striatal MSNs

**Figure 1:**
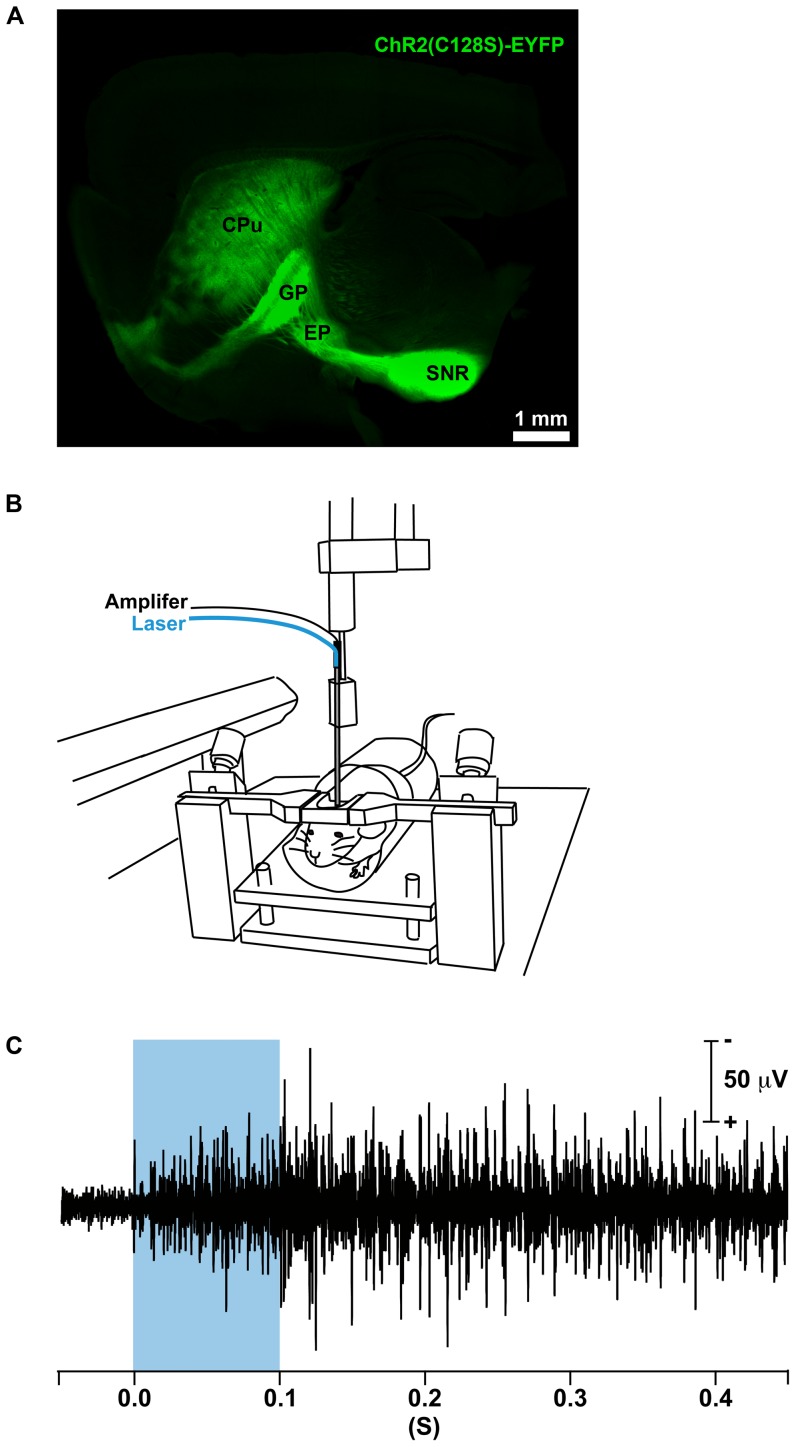
In vivo optical and physiological system for control of striatal MSNs in mice. (A) A sagittal section of the mouse brain showing selective expression of ChR2(C128S) in striatal MSNs as visualized by enhanced yellow fluorescent protein (EYFP) signals. Strong fluorescence was observed in the caudate putamen (CPu) as well as the targets of striatal MSNs such as the external segment of the globus pallidus (GP), the entopeduncular nucleus (EP) and the substantia nigra pars reticulata (SNR). (B) Schematic of the electrophysiological set-up used for in vivo photostimulation and electrophysiological recordings in awake mice. (C) A representative electrophysiological recording from the striatum. Photostimulation (a single 100-ms pulse, represented by a blue rectangle) in the striatum evoked neuronal excitation. The excitation lasted more than 1 minute (data not shown). Scale bar: (A) 1 mm.

To drive cell type-specific expression of a highly light-gated channelrhodopsin-2 (ChR2) variant ChR2(C128S) [12] we took advantage of the tetracycline-controlled transcriptional activator (tTA) system. Previously it was demonstrated that tTA is stably expressed in almost all striatal MSNs of PDE10A2-tTA mice [13]. Recently the tetO-ChR2(C128S)-EYFP BAC transgenic mouse line has been established in which the tTA-dependent promoter (tetO) drives the expression of ChR2(C128S) [14]. The above two lines were crossed to achieve striatal MSN-specific strong expression of ChR2(C128S). In the compound heterozygous mice strong fluorescent signals spanned the whole projection area of striatal MSNs including the caudate putamen (CPu), the fibers of striatal MSNs and their projection areas including the external segment of the globus pallidus (GP), the entopeduncular nucleus (EP) and the substantia nigra pars reticulata (SNR) (Figure 1A). We recorded extracellular neuronal activity of striatal neurons before and after illuminating with a single 100-ms pulse of blue laser (473 nm) in awake mice (Figure 1B, C). The laser illumination evoked prolonged excitation which lasted after termination of the photostimulation (Figure 1C). In our experiment, we used only blue light to modulate neuronal activity. Therefore, the excitation after a single pulse of photostimulation lasted more than 1 min (data not shown).

**Figure 2:**
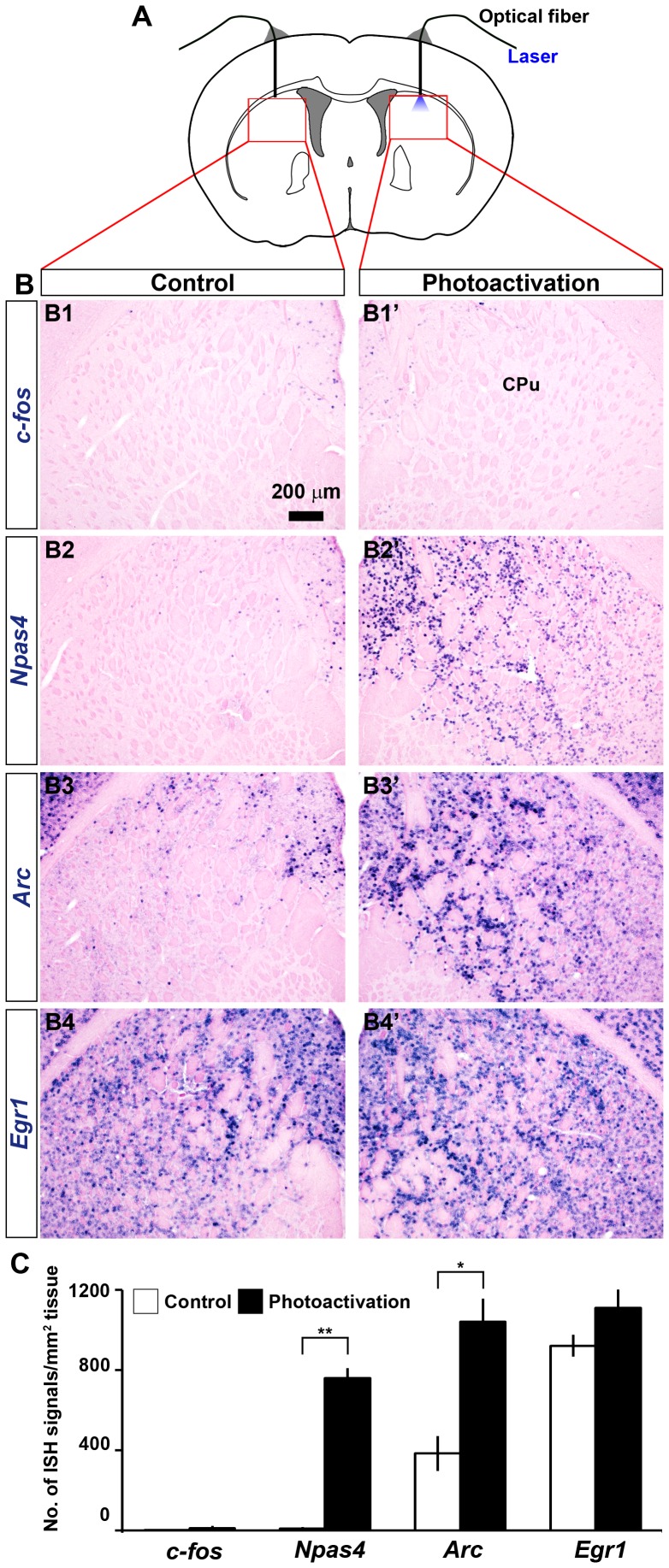
Identification of photoactivated neurons by IEG tracing. (A) Schematic used for in vivo photostimulation and histology in the mouse striatum. Blue light stimulation was given to the left striatum and the right striatum was used as the sham-treated control. Boxed areas indicate approximate striatal regions shown in B. (B) Representative images of coronal tissue sections from mice which received optogenetic stimulation in the left striatum showing ISH signals of *c-fos* (B1, B1'), *Npas4* (B2, B2'), *Arc* (B3, B3') and *Egr1* (B4, B4'). CPu, Caudate putamen. (C) Quantification of *c-fos*, *Npas4*, *Arc* and *Egr1* mRNA signals in the striatum after light stimulation. Induction of *c-fos* was not observed, whereas, a robust increase in *Npas4* mRNA signals appeared in the left striatum which received optogenetic stimulation. Although *Arc* was induced by photostimulation, the expression level was relatively high in the contralateral striatum. Any induction of *Egr1* was not apparent after illumination. Data represent mean ± SEM. ** Difference between groups was highly significant (p≤0.01), * Difference between groups was significant (p≤0.05). Scale bar: (B) 200 µm.

To perform in vivo optogenetic manipulation in freely moving animals we implanted optical fibers in the mouse brain targeting the striatum (Figure 2A). After recovery from implantation procedure mice were kept in their home cages where they did not show any altered behaviors. When awake mice in home cages were given blue light (a single 500-ms pulse) to illuminate the striatum in one hemisphere, animals displayed complex behaviors, including ipsilateral rotations (Sano and Tanaka, manuscript in preparation). These results indicated that light illumination reliably activated the striatal MSNs and the optogenetic modulation of neuronal excitability was sufficient to trigger recognizable behavioral responses in freely moving mice.

### Tracing photoactivated neurons

We looked for a selective increase in expression of IEGs in the ipsilateral striatum after 10 minutes of ChR2(C128S)-mediated unilateral photoactivation in the PDE10A2-tTA mice (Figure 2A). Any apparent induction of *c-fos*, the most widely used activity marker, was not observed in the striatum in our experimental conditions (Figure 2B1, B1', C). Therefore, it was necessary to check whether the striatum in the ChR2(C128S)-expressing mice can respond normally to known inducers of IEGs. We found that acute administration of methamphetamine (2 mg/kg, intraperitoneal injection, single dose), which is known to induce IEG expression in the striatum [15]–[18], significantly upregulated *c-fos* expression in the striatum of the ChR2(C128S)-expressing transgenic mice and the expression pattern was apparently similar to that of wild type mice (Figure S1). Additionally, our preliminary observation did not indicate any noticeable behavioral difference between the wild type and the transgenic mice without or with methamphetamine treatment (data not shown). Together, it seemed that the striatal functions were intact in the ChR2(C128S)-expressing PDE10A2-tTA line.

Subsequently, we expanded the screening using a number of IEGs which were found to be induced by brain activities in previous studies. We found strong signals of *Arc* mRNAs in the illuminated striatum (Figure 2B3') in addition to very dense signals in other brain regions including the cerebral cortex. Nonetheless, a considerable level of *Arc* mRNA expression was observed also in the contralateral side (Figure 2B3, C). Similarly, *Junb* expression was only slightly higher in the ipsilateral striatum compared to the contralateral striatum (Data not shown). We found that *Egr1* expression was not suitable for tracing photoactivation of striatal MSNs since dense mRNA signals appeared in both the control and the illuminated hemispheres (Figure 2B4, B4', C). In addition, any selective induction was absent for other IEGs such as *Fosb*, *Egr3* and *Jun* (Data not shown). In stark contrast, another neuronal IEG, the basic helix-loop-helix (bHLH)-PAS transcription factor *Npas4*, was robustly induced specifically in the ipsilateral striatum following photoactivation of striatal MSNs (Figure 2B2', C). Signals for *Npas4* mRNAs were almost absent in the contralateral sham-treated striatum (Figure 2B2, C) and in the striatum of the mice in which optical fibers were implanted in both hemispheres but no laser illumination was done (Figure S2).

**Figure 3:**
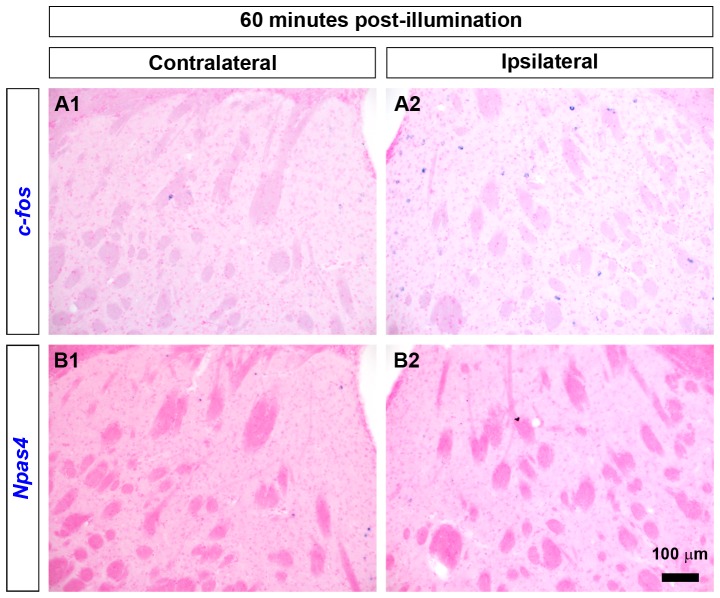
Expression of *c-fos* and *Npas4* in the striatum after 60 minutes of illumination. (A1–B2) No significant expression of either *c-fos* (A1, A2) or *Npas4* (B1, B2) was observed in the striatum after 60 minutes of ChR2(C128S)-mediated activation of MSNs. Scale bar: 100 µm.

We also checked IEG induction with an extended time window, 60 minutes after illumination. In contrast to 10 minutes post-illumination delay, there was almost no ISH signal of *Npas4* mRNA in the striatum after 60 minutes of photoactivation (Figure 3). These data were consistent with previous studies in respect to the quick and transient induction of *Npas4* [19], [20].

In some cases we observed induction of both *Npas4* (see later) and *c-fos* (data not shown) in the ipsilateral cortex after unilateral optical illumination of the striatum. Such cortical IEG induction was absent in the double-instrumented sham-treated mice (Figure S2B1–C2). It is possible that the cortical IEG induction we found was secondary effects of striatal stimulation. Previous studies also reported significant induction of *c-fos* expression in the cortex after striatal stimulation and disinhibition of the thalamocortical pathways has been suggested as a possible mechanism [21]–[24].

**Figure 4:**
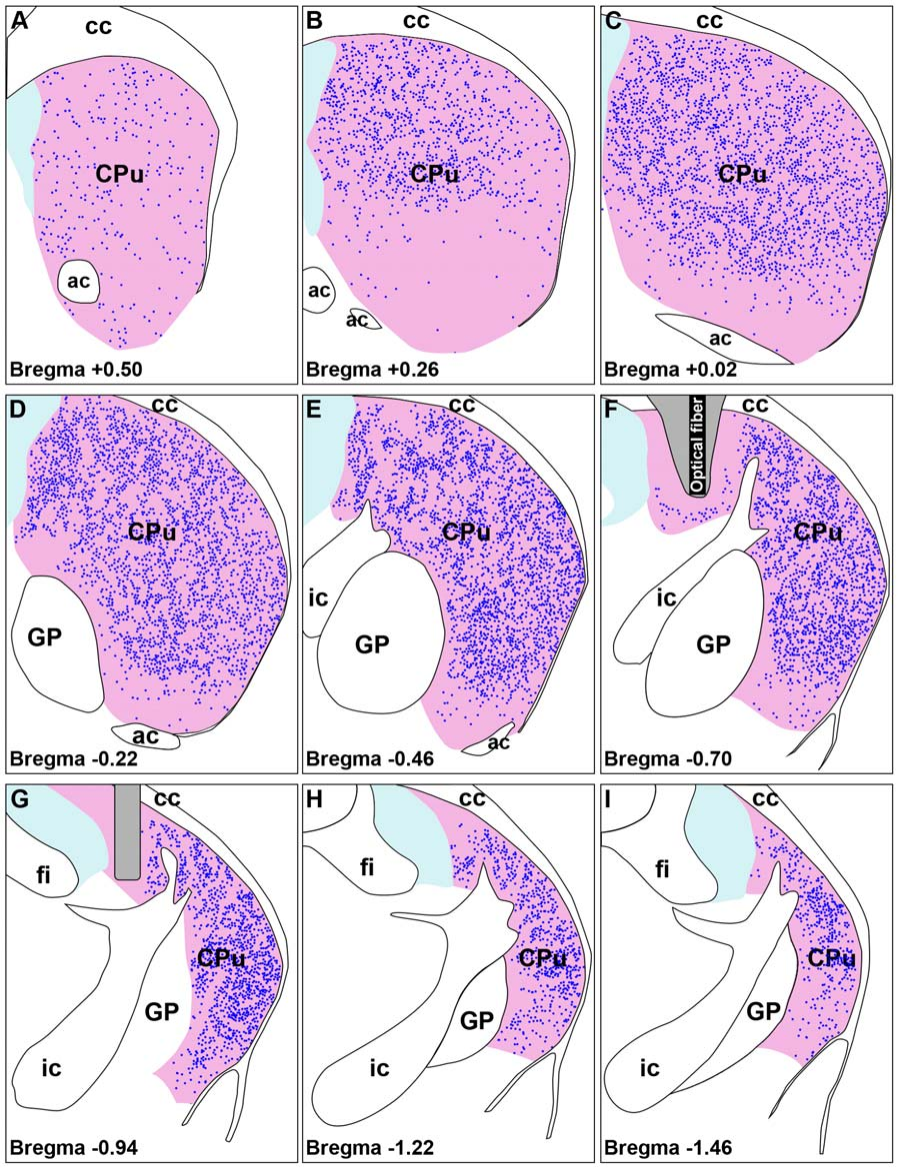
Distribution of *Npas4* ISH signals in the striatum after unilateral blue light illumination. Schematic diagrams of the striatum were constructed by superimposing images of stained coronal tissue sections on figures from a standard mouse brain atlas [27]. *Npas4* ISH signals in the illuminated striatum (CPu) are represented by blue dots. Signals outside the striatum are not shown. (A–I) Photoactivation-induced expression of *Npas4* in the mouse striatum is shown from bregma +0.50 mm to bregma −1.46 mm. The location of the optical fiber is depicted in F. The areas adjacent to the optical fiber appear in gray shade (F, G). (D–H) Strong *Npas4* induction took place in almost the entire extent of the striatal region close to the optical fiber. (A–B) *Npas4* expression level was relatively weaker along the ventral part of the rostral striatum. ac, Anterior commissure; cc, Corpus callosum; CPu, Caudate putamen; fi, Fimbria of the hippocampus; GP, Globus pallidus; ic, Internal capsule.

**Figure 5:**
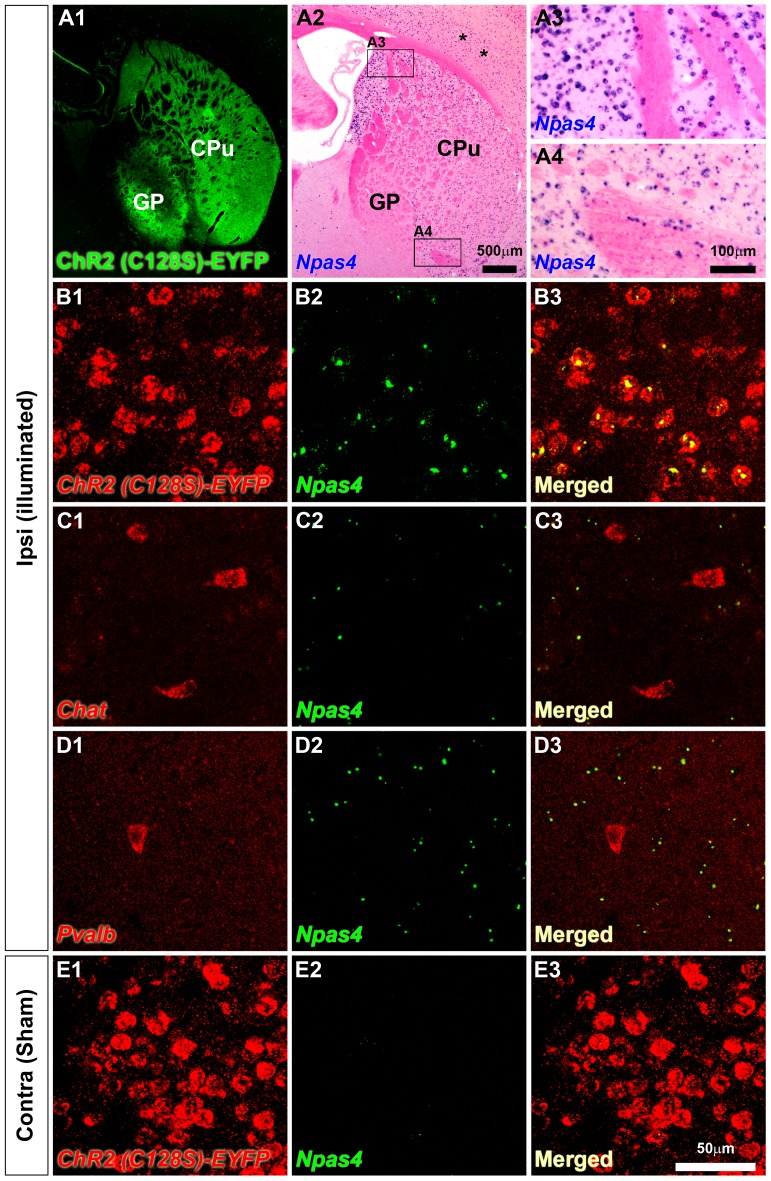
Optical stimulation-induced *Npas4* expression was mostly limited to the ChR2(C128S) expressing MSNs. (A1) A representative fluorescent image of coronal tissue sections indicates strong expression of the transgene (*ChR2(C128S)-EYFP*) in the striatum. (A2) After unilateral light stimulation *Npas4* mRNA expression was induced in the ipsilateral striatum (CPu, Caudate putamen). Note that *Npas4* induction was also observed in the cerebral cortex in some cases (asterisks, A2). Boxed areas in A2 are shown in higher magnification in A3 and A4. Induction of *Npas4* was observed from the dorsal (A3) to the ventral (A4) striatum. (B1–B3) Most of the striatal MSNs expressed the transgene *ChR2(C128S)-EYFP* (B1, red). After illumination *Npas4* (green) was expressed in the ipsilateral striatum (B2). Almost all *Npas4*-expressing cells were co-labeled with *ChR2(C128S)-EYFP* (B3). (C1–C3) Cholinergic interneurons were identified by *Chat* expression (C1, red). There was almost no co-labeling for *Npas4* (green) and *Chat* (red) (C2, C3). (D1–D3) *Npas4* (green) mRNA expression was not induced in *Pvalb* (red)-positive GABAergic interneurons. (E1–E3) Strong expression of *ChR2(C128S)-EYFP* (red) was observed in the contralateral sham-treated striatum (E1, E3) where *Npas4 (green)* expression was almost absent (E2, E3). CPu, Caudate putamen; GP, Globus pallidus. Scale bars: (A1, A2) 500 µm, (A3, A4) 100 µm, (B1–E3) 50 µm.

When propagating through a diffuse scattering media like brain tissue the incident light is attenuated resulting from a number of phenomena such as scattering, absorption and conical spreading [25]. Nonetheless, if the target volume is small (<1 mm^3^) light penetration is not a limiting factor and the entire target could be recruited [3], [25]. On the other hand, the mouse striatum is a relatively large structure comprising approximately 5–6% of the brain volume [26] and it extends approximately 4 mm and 3 mm along the rostrocaudal and dorsoventral axes, respectively [27]. Presumably, in vivo optogenetic modulation in mouse striatum will recruit a significant volume of tissue proximal to the tip of the optic fiber and may leave the distant regions mostly unaffected. In our experimental conditions light stimulation was applied to the dorsal striatum (Figure 4F, G) which has been implicated in motor control [28], [29]. Figure 4 shows expression of *Npas4* as observed in coronal sections of the brain hemisphere ipsilateral to optical illumination. *Npas4* induction took place along the entire mediolateral extent of the striatum near the tip of the optic fiber (Figure 4F). At the rostral striatum strong induction of *Npas4* appeared mostly in the dorsomedial part (Figure 4B–D) and expression level was low along the ventral striatum (Figure 4A, B). It is noteworthy that placement of the optical fiber (both location and orientation/angle of the tip) could influence the number of labeled neurons since the neurons which are distant from the probe may not be activated because of attenuation and conical spreading of the incident light [25]. Nevertheless, we observed reliable photostimulation in our experimental set up and it appeared that optical illumination recruited a substantial volume of striatum along the rostrocaudal, mediolateral and dorsoventral axes as revealed by the selective induction of *Npas4* mRNA expression (Figure 4, Figure 5A1–A4).

Major components of the basal ganglia neuronal network are MSNs, the most abundant cell type in the striatum, and there are several types of interneurons such as cholinergic interneurons and GABAergic interneurons [30]–[32]. We performed double fluorescent ISH to know which cell types in the striatum expressed *Npas4* after optical stimulation. In the illuminated striatum almost all *Npas4*-positive cells were double-positive for *Npas4* and *ChR2(C128S)-EYFP* (Figure 5A1–B3). Cholinergic interneurons, also known as tonically active neurons (TANs), exhibit spontaneous firing and are synaptically connected with MSNs [33], [34]. We used choline acetyltransferase (*Chat*) as a marker for cholinergic interneurons. It seemed that in cholinergic interneurons induction of *Npas4* was minimal, if any, since none of the *Chat* positive cells (56 cells examined using confocal microscopy) was co-labeled with *Npas4* in the striatum (Figure 5C1–C3). Seemingly, *Npas4* induction was also absent in parvalbumin (*Pvalb*, also known as PV)-positive GABAergic interneurons as we did not observe any *Pvalb* and *Npas4* double-positive cells (14 cells examined) in the illuminated striatum (Figure 5D1–D3). Therefore, these data indicated that *Npas4* mRNA expression was induced by photostimulation mostly in the striatal MSNs which expressed ChR2(C128S)-EYFP (Figure 5).

## Discussion

Despite the fact that striatal MSNs have been strongly implicated in motor control, motivation and habit formation as well as neurological disorders like PD, Huntington's disease (HD), schizophrenia and addiction [35]–[37], a comprehensive understanding of their biological functions remains a major challenge. In addition to the classical anatomical studies [38], [39], recent technical advances in neuroscience have started unveiling the intricate connections and functional significance of MSNs [34], [40], [41]. Nonetheless, a major obstacle in studying the functions of striatum is the presence of heterogeneous population of MSNs which are intermingled in the basal ganglia networks [42].

Although intervening striatal activity through optogenetic manipulation can be advantageous in above regards, a precise identification of activated neurons would be necessary to interpret the cellular and behavioral changes which are ensued. In mice, induction of *c-fos* mRNA or Fos immunoreactivity has been used as a proxy for photoactivation of neurons in different brain regions such as the hypothalamus [5], the cerebral cortex [4] and the striatum [40]. Unexpectedly, we did not observe *c-fos* induction after in vivo unilateral stimulation of the dorsal striatum (Figure 2). The channelrhodopsin variant ChR2 was used in those previous studies and multiple light pulses were delivered over several minutes whereas we used the channelrhodopsin variant ChR2(C128S) and applied a single 500-ms pulse of blue light. ChR2 is photodepolarized rapidly and has a moderate channel closing rate while ChR2(C128S) has an increased light sensitivity with a slow off-kinetics [12], [43], [44]. Consequently, we were convinced that a number of experimental parameters such as the channelrhodopsin variant, number of light pulses and duration of stimulation could account for the absence of *c-fos* mRNA induction in our study. We also found that most of the commonly used IEGs were unsuitable to trace photoactivation in the mouse striatum. We then analyzed expression of *Npas4* which was previously shown to be induced by in vivo sensory stimulation in the visual cortex [20] and has been implicated in learning and memory [45], [46]. It has been reported that *Npas4* is expressed in different brain regions, selectively in neurons and predominantly in excitatory neurons [19]. We found that in the striatum *Npas4* expression was very low or absent at the basal condition (Figure 2B2, Figure S1). Interestingly, in vivo optical illumination in freely moving mice differentially induced *Npas4* expression specifically in the ipsilateral striatum (Figure 2B2').

Can we also expect *Npas4* induction following ChR2(C128S)-mediated photostimulation in other neuronal cell types or other brain regions? Illumination to ChR2(C128S)-expressing hippocampal CA1 neurons resulted in induction of *Npas4* [KFT unpublished data] in addition to *c-fos* induction [14]. However, induction of *Npas4* was not apparent in raphe neurons where ChR2(C128S) was expressed by taking advantage of the tryptophan hydroxylase 2 (Tph2)-tTA line [KFT, unpublished data]. Thus, induction of a particular IEG, such as *Npas4* and *c-fos*, following optical activation should be assessed for the neuronal cell type of interest.

Differential IEG induction following a given stimulus has also been demonstrated in previous studies [8], [11], [20]. Although expression of *Npas4* was found to be selectively induced by membrane depolarization and Ca^2+^ influx, *Npas4* was not induced by several neurotrophic factors like BDNF and NT3 which readily induce other transcription factors such as *c-fos* and *Arc* [19], [20]. It was found that after contextual fear conditioning (CFC) *Npas4* induction took place as early as 5 minutes after training, much earlier than *c-fos* induction and the authors suggested that the pathways which induce *Npas4* expression could be different from those for other IEGs [20]. Therefore, it is possible that in our study optical activation of ChR2(C128S)-expressing striatal MSNs triggered cellular pathways which were sufficient for induction of *Npas4* but not for other IEGs like *c-fos*. Taken together, our results suggest that *Npas4* can be a suitable tracer for identification of photoactivated MSNs at the cellular level in the mouse striatum considering the very low basal expression and a rapid and robust induction following stimulation.

## Materials and Methods

### Mice

PDE10A2-tTA mice were obtained from RIKEN BRC Bank (RBRC No. RBRC02317, Strain B6.129-Pde10a2<tm1(tTA)Yok>) [13]. The tetO-ChR2(C128S)-EYFP BAC transgenic mice were crossed to PDE10A2-tTA mice to generate compound heterozygous mice [14]. Animals were kept under regulated air conditions (23°C±1°C) and a 12∶12 hours light-dark cycle throughout the experiments. Food and water were available ad libitum. Two to three mice were analyzed for each condition. All animal procedures were approved by the Animal Research Committees of the National Institute for Physiological Sciences, Keio University and Niigata University.

### In vivo optical stimulation and electrophysiological recording of striatal neurons

To fix the head of the awake mouse in a stereotaxic apparatus, a small U-frame head holder was mounted on the head as reported previously [47]. Each mouse was anesthetized with ketamine hydrochloride (100 mg/kg body weight, i.p.) and xylazine hydrochloride (5 mg/kg body weight, i.p.) and fixed in a conventional stereotaxic apparatus (Narishige Scientific Instrument, Tokyo, Japan). The skull was widely exposed, and periosteum and blood on the skull were removed completely. The exposed skull was completely covered with bone adhesive resin (BISTITE II, Tokuyama, Tokyo, Japan) and acrylic resin (UNIFAST II, GC Corporation, Tokyo, Japan), and then a small U-frame head holder for head fixation was mounted and fixed with acrylic resin on the head of the mouse. After recovery from the first surgery (2 or 3 days later), the mouse was positioned in a stereotaxic apparatus with its head restrained using the U-frame head holder under light anesthesia with ketamine hydrochloride (50–100 mg/kg body weight, i.p.). A part of the skull in one hemisphere was removed to access the striatum.

After full recovery from the second surgery, the mouse was positioned in a stereotaxic apparatus with its head restrained using a U-frame head holder in the awake condition. For recording neural activity while illuminating with blue light, an electrode assembly consisting of a glass-coated Elgiloy microelectrode (0.5–1.0 MΩ at 1 kHz) and a 50 µm diameter optical fiber (CeramOptec Industries, East Longmeadow, MA, USA), was inserted perpendicularly into the brain through the dura mater using a hydraulic microdrive (Narishige Scientific Instrument, Tokyo, Japan). A blue laser (50 mW, CrystaLaser, Reno, NV, USA) was coupled to the optical fiber. The laser power was ≈40 mW at the fiber tip. The laser was controlled via TTL pulses driven by a stimulator (Nihon Kohden, Tokyo, Japan). The target area was 0.0–0.5 mm anterior and 2.0–2.2 mm lateral to bregma and 2.5–4.0 mm deep from the brain surface for the striatum [27]. Signals from the electrode were amplified, filtered (0.3–10 kHz), and sampled at 50 kHz using a computer.

### In vivo optical stimulation in freely moving mice

Each mouse was anesthetized with ketamine hydrochloride and xylazine hydrochloride and fixed in a conventional stereotaxic apparatus (David Kopf, CA, USA). A plastic optical fiber (ESKA, Mitsubishi Rayon, Tokyo, Japan, 0.5 mm diameter) was inserted in each cerebral hemisphere above the dorsal striatum. The tip of the fiber located approximately at 0.7 mm posterior and 2.0 mm lateral to bregma, and 2.0 mm deep from the skull. The fiber was fixed on the skull using Aron Alpha (Toagosei Co., LTD., Tokyo, Japan). The mice were allowed to recover for at least one week after fiber implantation. For optical stimulation of striatal neurons, a single 500-ms illumination (6.7 mW/mm^2^ at the fiber tip) was given to the left striatum in the home cage. No optical stimulation was given to the right striatum and this served as the sham-treated control. Mice were anesthetized with ketamine hydrochloride and xylazine hydrochloride after 5 minutes of optical stimulation and perfused transcardially after additional 5 minutes.

### In situ hybridization

*In situ* hybridization was performed as described previously [48] using DIG-labeled riboprobes (Table S1). Briefly, 20-µm sections were prepared from frozen mouse brain samples. Sections were fixed in 4% PFA, digested with Proteinase K (1 µg/ml), acetylated and then hybridized with DIG-labeled riboprobes overnight at 65°C. DIG-labeled RNA hybrids were reacted with an alkaline phosphatase-conjugated anti-DIG antibody (1∶2000, Roche) overnight at 4°C. Sections were washed in MABT (100 mM Maleic acid, 150 mM NaCl, 0.1% Tween 20) and then in alkaline phosphatase buffer (100 mM NaCl, 100 mM Tris-HCl, pH 9.5, 50 mM MgCl_2_, 0.1% Tween 20, 5 mM Levamisole). Tissue sections were treated with NBT/BCIP (Roche) mixture at room temperature in dark for color development. After ISH staining, the sections were counterstained by nuclear fast red.

### Double fluorescent in situ hybridization (Double FISH)

Procedures for double FISH were adopted from a previous study [14]. In brief, frozen tissue sections were hybridized with FITC-labeled *Npas4* cRNA probe and DIG-labeled *GFP*, *Chat* or *Pvalb* cRNA probes. After stringent washing sections were incubated with a peroxidase-conjugated anti-FITC antibody (Roche, 1∶200, 30 minutes at room temperature) and signals were visualized by FITC (TSA Plus Cyanine 3/Fluorescein System, PerkinElmer, Foster city, CA). Residual peroxidase activity was quenched by 2% H_2_O_2_ (30 minutes at room temperature). Samples were then incubated with a peroxidase-conjugated anti-DIG antibody (Roche, 1∶200, 60 minutes at room temperature) and signals were visualized by Cy3 (TSA Plus Cyanine 3/Fluorescein System, PerkinElmer). Confocal fluorescence images were captured using a laser scanning microscope system (LSM 710, Carl Zeiss Microimaging, Germany). It is notable that in the standard chromogenic *Npas4* ISH, very often we observed weaker perinuclear signals and stronger nucleolar signals (arrows, Figure S3). In Double FISH using TSA amplification system, we mostly observed strong dot-like *Npas4* signals.

### Quantification of ISH signals

Images of stained coronal sections of the mouse brain were captured with an Olympus microscope (BX53, Olympus, Tokyo, Japan) and digital camera system (DP72, Olympus). The cells which were positive for ISH signals were counted in the dorsolateral striatum from five coronal tissue sections (approximately ±0.5 mm bregma) from one representative animal. Student's t-test was performed to compare means. Difference between groups was considered highly significant when p≤0.01 and significant when p≤0.05.

## Supporting Information

Figure S1 **Methamphetamine induced IEG expression in the striatum of the mice which expressed ChR2(C128S) in MSNs.** (A1, A2) A single dose of methamphetamine (2 mg/kg, i.p.) significantly induced expression of *c-fos* mRNAs in the striatum in both the BAC transgenic (A1) and the wild type mice (A2). (B1, B2) In both the transgenic (B1) and the wild type mice (B2), *Npas4* was slightly induced after the acute methamphetamine treatment. Scale bar: 100 µm.
(TIF)

Figure S2 ***Npas4*** **expression was almost absent in the double-instrumented, non-illuminated mice which expressed ChR2(C128S).** (A) Schematic diagram showing sham control experiments where optical fibers were implanted in both hemispheres but no illumination was given. (B1–C2) Sham operations did not induce expression of either *c-fos* (B1, B2) or *Npas4* (C1, C2) in the striatum of ChR2(C128S)-expressing mice. Scale bars: (B1, C1) 500 µm, (B2, C2) 50 µm.
(TIF)

Figure S3 **Subcellular distribution of** ***Npas4*** **ISH signals.** (A, B) *Npas4* ISH signals in the striatum of mice after 10 minutes of unilateral optical stimulation. In the standard chromogenic *Npas4* ISH, weaker perinuclear signals (arrowheads, B) and stronger nucleolar signals (arrows, B) were observed. The boxed area in A is shown in higher magnification in B. Scale bars: (A) 100 µm, (B) 50 µm.
(TIF)

Table S1 **Information on ISH probes used in this study.**
(DOC)
