# Supplementary figures and images for: Identification of Optogenetically Activated Striatal Medium Spiny Neurons by Npas4 Expression
Source: PLoS One. 2012 Dec 26;7(12):e52783. doi: 10.1371/journal.pone.0052783 (PMC3530472; doi:10.1371/journal.pone.0052783)

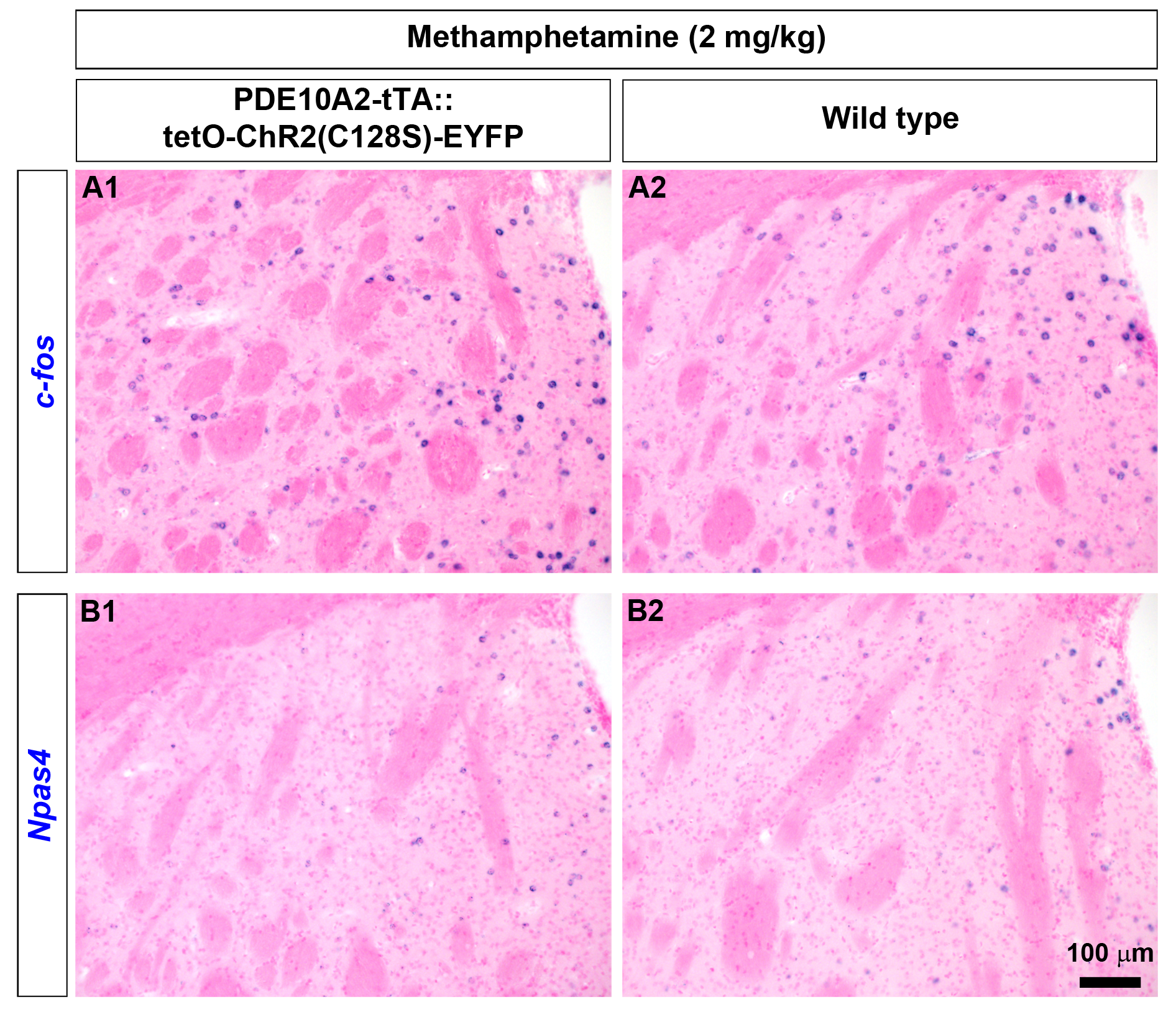

Supplement: Figure S1 — Methamphetamine induced IEG expression in the striatum of the mice which expressed ChR2(C128S) in MSNs. (A1, A2) A single dose of methamphetamine (2 mg/kg, i.p.) significantly induced expression of c-fos mRNAs in the striatum in both the BAC transgenic (A1) and the wild type mice (A2). (B1, B2) In both the transgenic (B1) and the wild type mice (B2), Npas4 was slightly induced after the acute methamphetamine treatment. Scale bar: 100 µm. (TIF) [file pone.0052783.s001.tif]

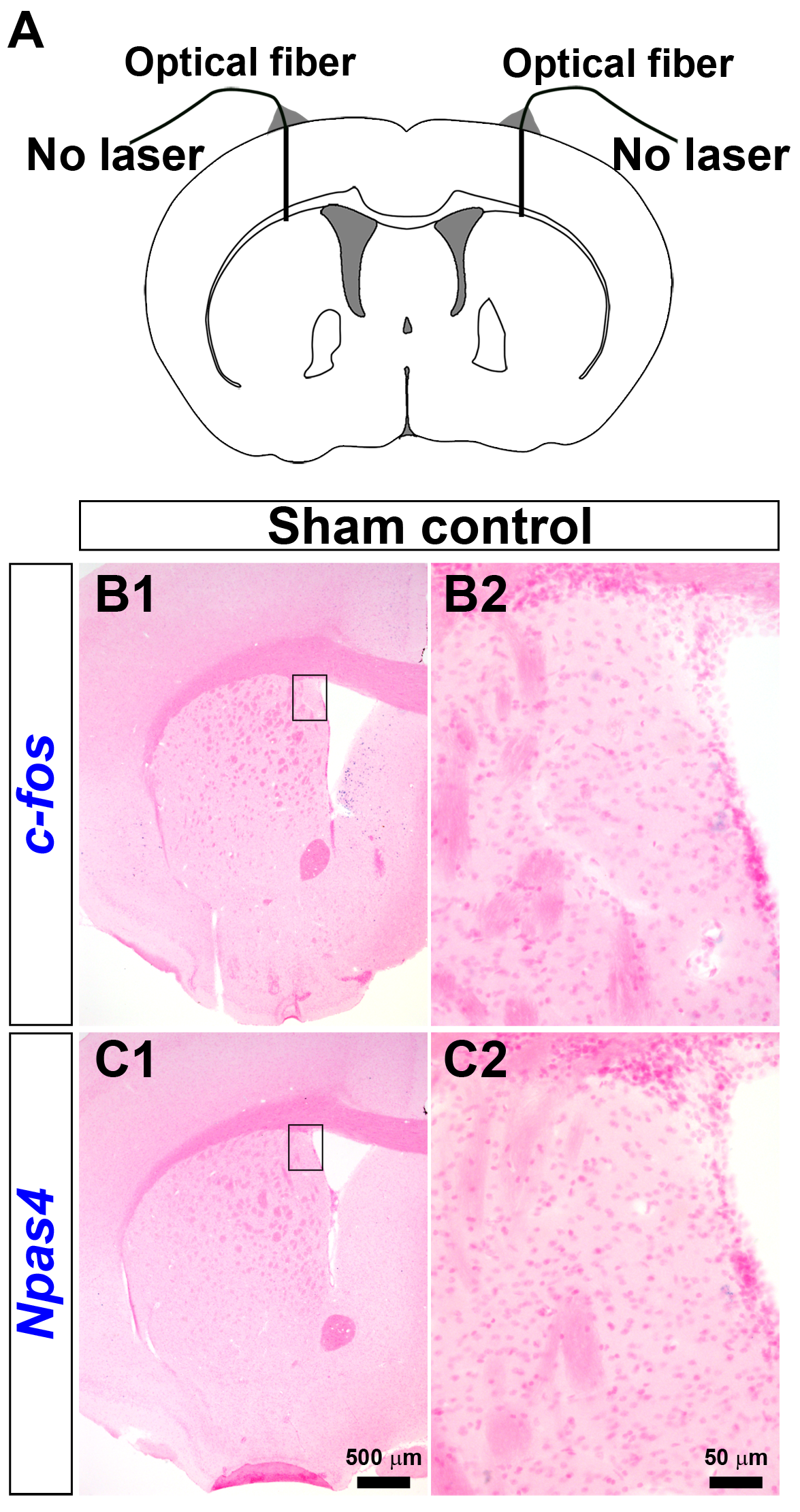

Supplement: Figure S2 — Npas4 expression was almost absent in the double-instrumented, non-illuminated mice which expressed ChR2(C128S). (A) Schematic diagram showing sham control experiments where optical fibers were implanted in both hemispheres but no illumination was given. (B1–C2) Sham operations did not induce expression of either c-fos (B1, B2) or Npas4 (C1, C2) in the striatum of ChR2(C128S)-expressing mice. Scale bars: (B1, C1) 500 µm, (B2, C2) 50 µm. (TIF) [file pone.0052783.s002.tif]

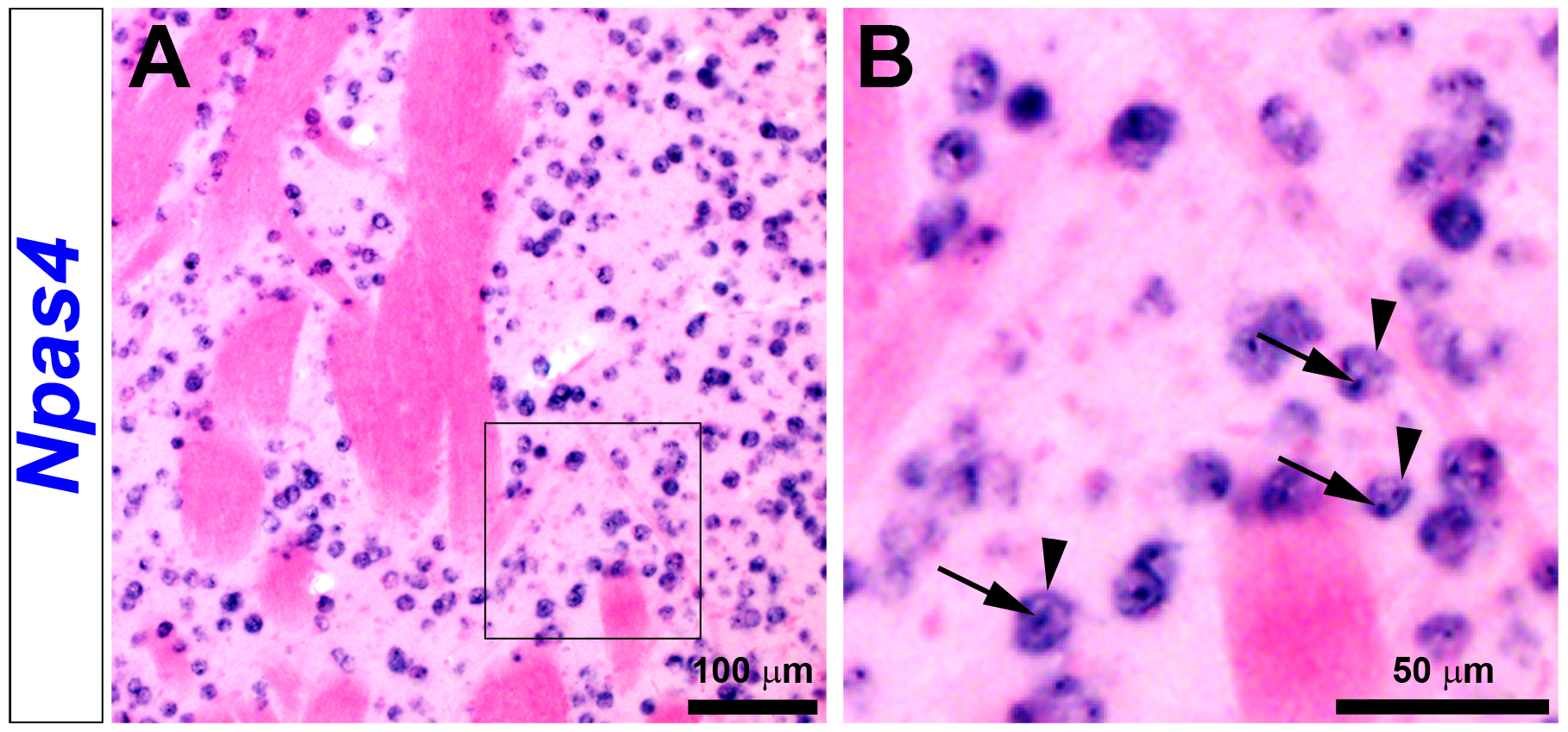

Supplement: Figure S3 — Subcellular distribution of Npas4 ISH signals. (A, B) Npas4 ISH signals in the striatum of mice after 10 minutes of unilateral optical stimulation. In the standard chromogenic Npas4 ISH, weaker perinuclear signals (arrowheads, B) and stronger nucleolar signals (arrows, B) were observed. The boxed area in A is shown in higher magnification in B. Scale bars: (A) 100 µm, (B) 50 µm. (TIF) [file pone.0052783.s003.tif]
