# Supplementary material for: Identification of Optogenetically Activated Striatal Medium Spiny Neurons by Npas4 Expression
Source: PLoS One. 2012 Dec 26;7(12):e52783. doi: 10.1371/journal.pone.0052783 (PMC3530472; doi:10.1371/journal.pone.0052783)
Supplement: Table S1 — Information on ISH probes used in this study. (DOC) [file pone.0052783.s004.doc]

**Table S1: Information on ISH probes**

| **Gene symbol &**  Genbank accession number | **Name**  **Synonyms** | **Forward primer**  **Reverse primer** | **Product (bp)** | **Vector** | **Restriction enzyme for AS probe** | **RNA polymerase for AS probe** | **Remarks/Reference** |
| --- | --- | --- | --- | --- | --- | --- | --- |
| ***Arc***  AF162777 | **activity regulated cytoskeletal-associated protein**  Arc3.1, arg 3.1 | 5’-CGCAGCACCGACGACCAGAT-3’  5’-GACCAGGGCAGACAGATGAG-3’ | 1449 | p123T | BamHI | T7 |  |
| ***c-fos***  BC029814 | **FBJ osteosarcoma oncogene**  Fos, cFos | None | ~2107 | pCMV-SPORT6 | EcoRI | T7 | EST clone |
| ***Chat***  NM_009891 | **choline acetyltransferase** | See reference |  | pBluescript SK(-) | EcoRI | T7 | Ref. Dev Biol 293: 348-357, 2006. |
| ***Egr1***  NM_007913 | **early growth response 1**  Krox-24, NGFI-A, Zif268 | None | ~3072 | pCMV-SPORT6 | EcoRI | T7 | EST clone |
| ***Egr3***  NM_018781 | **early growth response 3**  Pilot | 5’-CAATCTGTACCCCGAGGAGA-3’  5’-CCACAGAACTCACAGGCAAA-3’ | 951 | p123T | BamHI | T7 |  |
| ***Fosb***  NM_008036 | **FBJ osteosarcoma oncogene B** | 5’-CGGTCTCGGGGAAATGCCCG-3’  5’-GCGAGTTCAGCGGGTCGGAC-3’ | 863 | pCR II | EcoRV | Sp6 |  |
| ***GFP*** (Used for detection of ChR2(C128S)-EYFP). |  | See remarks | ~741 | pcDNA3 | BamHI | Sp6 | BamHI-NotI fragment from pEGFP-1 was inserted in pcDNA3. |
| ***Jun***  BC094032 | **Jun oncogene**  c-jun, Junc | None | ~1866 | pCMV-Sport6 | EcoRV | T7 | EST clone |
| ***Junb***  BC003790 | **Jun-B oncogene** | None | ~1780 | pCMV-Sport6 | EcoRV | T7 | EST clone |
| ***Npas4***  NM_153553 | **neuronal PAS domain protein 4**  LE-PAS, Npas4, Nxf | 5’-TCTCACTGTGCGCCAGCAGC-3’  5’-CCACGCCCTGAGCCAACTGG-3’ | 1556 | pCR II | BamHI | T7 |  |
| ***Nr4a1***  NM_010444 | **nuclear receptor subfamily 4, group A, member 1**  NGFI-B, Nur77 | 5’-GCGGAACCGCTGCCAGTTCT-3’  5’-GTTGGAGGCTCGCCCAGCTG-3’ | 1233 | pCR II | BamHI | T7 |  |
| ***Nr4a3*** NM_015743 | **nuclear receptor subfamily 4, group A, member 3**  MINOR, Nor1, TEC | See reference | 393 | pBluescript SK(+) | HindIII | T3 | From Dr Levesque, Université de Montréal  Ref. J Pharmacol Exp Ther 313: 460-473, 2005. |
| ***Pvalb*** NM_013645 | **Parvalbumin**  Parv, PV, Pva | See reference | 563 | pCRII | BamHI | T7 | Ref. Neurosci Res 63: 213-223, 2009. |
